# Proteome level analysis of drug-resistant *Prevotella melaninogenica* for the identification of novel therapeutic candidates

**DOI:** 10.3389/fmicb.2023.1271798

**Published:** 2023-09-22

**Authors:** Mohibullah Shah, Amna Anwar, Aqsa Qasim, Samavia Jaan, Asifa Sarfraz, Riaz Ullah, Essam A. Ali, Umar Nishan, Muhammad Shehroz, Aqal Zaman, Suvash Chandra Ojha

**Affiliations:** ^1^Department of Biochemistry, Bahauddin Zakariya University, Multan, Pakistan; ^2^Medicinal Aromatic and Poisonous Plants Research Center, College of Pharmacy King Saud University, Riyadh, Saudi Arabia; ^3^Department of Pharmaceutical Chemistry, College of Pharmacy, King Saud University, Riyadh, Saudi Arabia; ^4^Department of Chemistry, Kohat University of Science and Technology, Kohat, Pakistan; ^5^Department of Bioinformatics, Kohsar University Murree, Murree, Pakistan; ^6^Department of Microbiology and Molecular Genetics, Bahauddin Zakariya University, Multan, Pakistan; ^7^Department of Infectious Diseases, The Affiliated Hospital of Southwest Medical University, Luzhou, China

**Keywords:** *Prevotella melaninogenica*, drug target, epitope, peptide vaccine, immunoinformatics

## Abstract

The management of infectious diseases has become more critical due to the development of novel pathogenic strains with enhanced resistance. *Prevotella melaninogenica*, a gram-negative bacterium, was found to be involved in various infections of the respiratory tract, aerodigestive tract, and gastrointestinal tract. The need to explore novel drug and vaccine targets against this pathogen was triggered by the emergence of antimicrobial resistance against reported antibiotics to combat *P. melaninogenica* infections. The study involves core genes acquired from 14 complete *P. melaninogenica* strain genome sequences, where promiscuous drug and vaccine candidates were explored by state-of-the-art subtractive proteomics and reverse vaccinology approaches. A stringent bioinformatics analysis enlisted 18 targets as novel, essential, and non-homologous to humans and having druggability potential. Moreover, the extracellular and outer membrane proteins were subjected to antigenicity, allergenicity, and physicochemical analysis for the identification of the candidate proteins to design multi-epitope vaccines. Two candidate proteins (ADK95685.1 and ADK97014.1) were selected as the best target for the designing of a vaccine construct. Lead B- and T-cell overlapped epitopes were joined to generate potential chimeric vaccine constructs in combination with adjuvants and linkers. Finally, a prioritized vaccine construct was found to have stable interactions with the human immune cell receptors as confirmed by molecular docking and MD simulation studies. The vaccine construct was found to have cloning and expression ability in the bacterial cloning system. Immune simulation ensured the elicitation of significant immune responses against the designed vaccine. In conclusion, our study reported novel drug and vaccine targets and designed a multi-epitope vaccine against the *P. melaninogenica* infection. Further experimental validation will help open new avenues in the treatment of this multi-drug-resistant pathogen.

## Introduction

*Prevotella melaninogenica* is a gram-negative, anaerobic, black-pigmented, and short rod-shaped bacterium. It is mainly involved in polymicrobial infections spreading throughout the body and mainly in the respiratory tract. Patients suffering from cystic fibrosis are diagnosed with *Prevotella* species in their respiratory tract (Sherrard et al., 2014). *P. melaninogenica* can be detected in saliva and at the dorsal and lateral sites of the tongue, which contain a high proportion. Mucosal surfaces of the aerodigestive tract, i.e., lungs, are majorly colonized by *Prevotella* species (Könönen and Gursoy, 2022). The oral cavity of humans is also a site of *P. melaninogenica* colonization. This species is involved in diseases of the gastrointestinal tract, acute and chronic ailments of the respiratory tract, and cancers of the digestive tract (Könönen and Gursoy, 2022). An inflammatory disease, oral lichen planus (OLP), is caused by *P. melaninogenica* in the oral mucosa with white striation lesions, repeated erosions, and pain (Zheng et al., 2022).

*P. melaninogenica* potentially leads to oral lichen planus (OLP) through different mechanisms. It uses T6SS protease to initiate the degradation of the epithelial barrier, which leads to dysfunction and an imbalance in surface flora (Kondo et al., 2018). *P. melaninogenica*, then, infiltrates the basal layer and lamina propria as target antigens, triggering recognition by innate immune cells such as macrophages and keratinocytes. Activation of NF-κB pathways by *P. melaninogenica* leads to the production of various cytokines and chemokines. This, in turn, results in the recruitment and infiltration of CD4+ and CD8+ T lymphocytes that attack epithelial keratinocytes, which causes the degeneration of the basal cell layer and leads to further impairment of the epithelial barrier. This destructive cycle leads to chronic infection and persistent inflammatory responses in OLP (Zheng et al., 2022).

The subgingival plaque represents the existence of *P. melaninogenica* in patients with periodontal diseases. The significance of periodontal abscesses is categorized by periodontal pathogens and black-pigmented microorganisms such as *P. melaninogenica* (He et al., 2013). Pro-inflammatory short-chain fatty acids can be produced due to the enhancement of bacterial pathogenicity in the lungs in the presence of these bacteria. This is one of the mechanisms that cause resistance to antibiotics. Resistance can be caused by resistance genes or the production of an enzyme (beta-lactamase). Resistance can also be developed by repetitive use or administration of high doses of antibiotics, as in patients with cystic fibrosis (Lamoureux et al., 2021).

There is an emerging trend toward developing resistance against tetracycline and penicillin among pigmented species. Beta-lactamase production causes resistance to penicillin (Troil-linde, 1999). A few antibiotics can still be used against *P. melaninogenica* such as metronidazole, clindamycin, imipenem, meropenem, and cefoxitin, but there is a risk of developing resistance against these antimicrobials in the near future (Troil-linde, 1999). Moreover, new therapeutic strategies are required to ensure the prevention of infections caused by *P. melaninogenica*.

The vaccine to combat *P. melaninogenica* infections is still not available. There is no information about the vaccine's development in the literature. This developed a need to design novel drug and vaccine targets that would be helpful in the near future. In this context, this study is aimed at identifying novel drug and vaccine targets against this bacterium to provide alternative potential therapeutic targets for the efficient action of antimicrobials and vaccine candidates (Qasim et al., 2023). The approach employed in this study is significant to minimize labor and focus on the development of vaccine candidates' predictions by utilizing bioinformatics tools.

## Materials and methods

**Figure 1:**
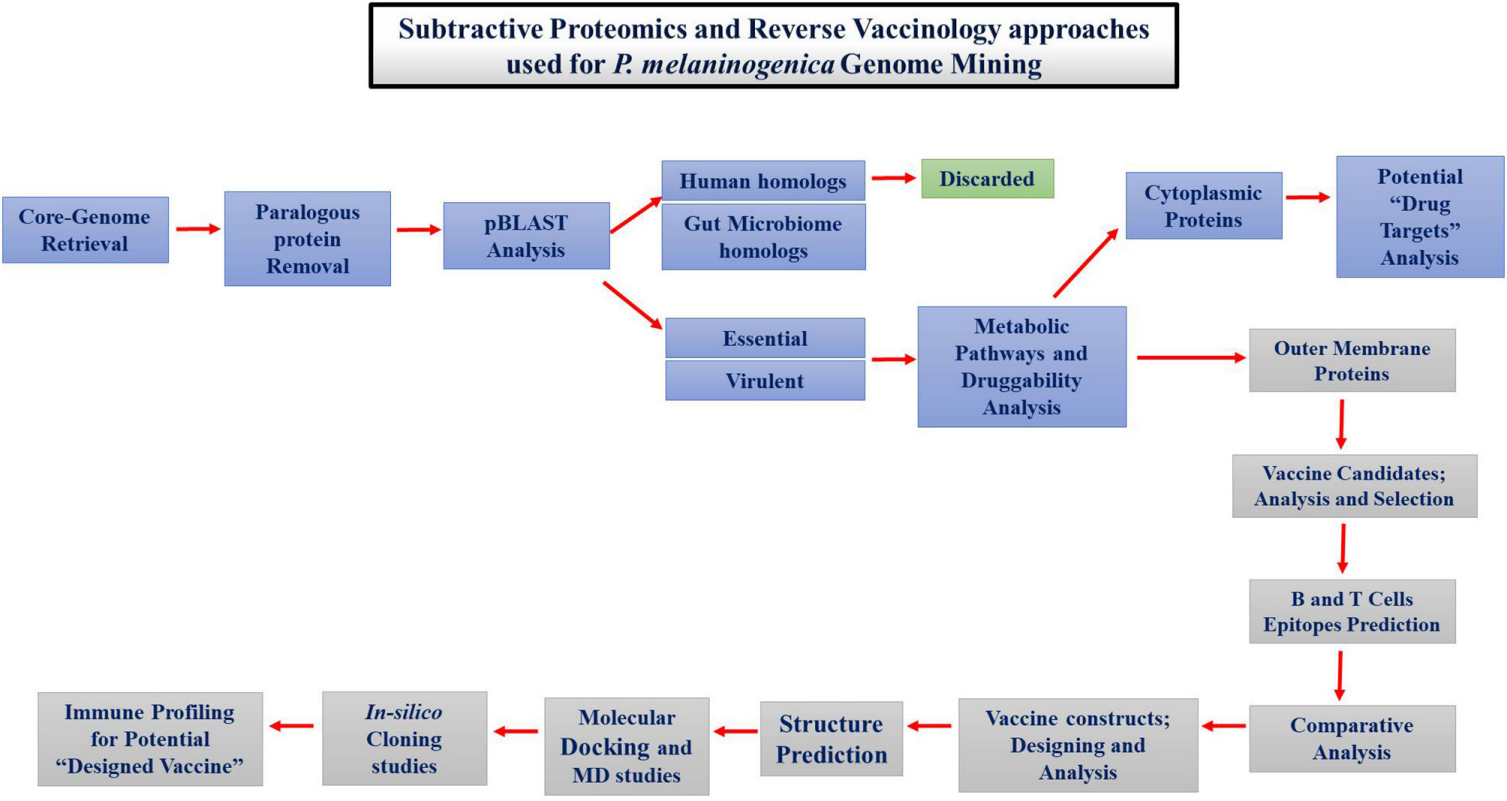
Schematic representation of approaches followed for therapeutic studies against *Prevotella melaninogenica* infection.

The proteins necessary for the survival of *P. melaninogenica* were determined by subtractive proteomics. Different tools and databases were used to find drug and vaccine targets, as shown in Figure 1.

### Retrieval of core gene sequences

The complete genome sequences of the 14 strains of *P. melaninogenica* were used to retrieve a core genome from the EDGAR tool version 2 (Blom et al., 2016). Clustering techniques were employed to identify duplicate or paralogous proteins *via* CD-HIT analysis (Li and Godzik, 2006), to remove redundancy. The cutoff value of 60% sequence similarity was kept as a threshold to obtain non-paralogous proteins, which were used for further analysis (Qasim et al., 2023). Alignment coverage was obtained by setting parameters at default.

### Determination of human non-homologs

Human host non-homologous proteins were determined by standalone BLASTp analysis, which was used for scanning against species non-paralogous proteins with E-value 1e-20, bitscore ≥100, percent identity ≥35, and query coverage ≥35 (Shah et al., 2021). The human proteome was downloaded from the UniProt database, comprising 1,076,164 proteins.

Further scanning of pathogen proteins was carried out against the human gut microbiome. The NCBI database was used to acquire a human gut microbiome consisting of 75,176 proteins. BLASTp was performed with criteria as follows: E-value 1e-4, bitscore ≥100, percent identity ≥50, and query coverage ≥35, as in our previous studies (Aslam et al., 2020; Shah et al., 2021; Jaan et al., 2022; Qasim et al., 2023). All the homologs were discarded.

### Identification of pathogen-essential proteins

After excluding gut microbiome homologs, essential proteins of the pathogen were determined by subjecting proteins to the database of essential genes (DEG) (http://tubic.tju.edu.cn/deg/) (Luo et al., 2021). DEG contains all the genes required for the survival of the pathogen. BLASTp was performed for scanning against DEG with cutoff parameters of E-value 1e-4 and bitscore ≥100 for the determination of *P. melaninogenica* essential genes.

### Identification of virulence proteins

The host defense mechanism is destroyed by the virulence factors of bacteria, which help to cause the disease through invasion, colonization, and adhesion. Four categories of virulence factors are included in the virulence factor database (VFDB) (Liu et al., 2019), which is used to identify virulence factors in a pathogen. From 25 pathogenic bacteria, offensive, defensive, non-specific, and virulence-related proteins are assembled in this database. Pathogen proteins were subjected to BLASTp analysis against VFDB with parameters, i.e., E-value 1e-4 and bitscore ≥100 (Qasim et al., 2023).

### Identification of antibiotic resistance and host–pathogen-interacting proteins

Databases were employed to identify resistant and host–pathogen-interacting proteins with criteria such as E-value 1e-4 and bitscore ≥100. There is an emerging trend toward developing resistance to available antibiotics among bacterial pathogens. Moreover, antibiotic-resistant genes were identified by the ARG-ANNOT database (Gupta et al., 2014), with selected criteria by subjecting pathogen proteins to BLASTp (Aslam et al., 2020). The host–pathogen interaction database (HPIDB v2.0) (Ammari et al., 2016) was used for the curation of host–pathogen protein–protein interactions, which were essential for the survival of a pathogen. Standalone BLASTp was used for the screening of interacting proteins against the HPIDB repository (Aslam et al., 2020).

### Metabolic pathway determination

Metabolic pathways of living organisms can be retrieved from the Kyoto Encyclopedia of Genes and Genomes (KEGG) database (Fatoba et al., 2021). Human non-homologous essential proteins were further subjected to KEGG for metabolic pathway determination. Functional annotation of proteins was provided by the KAAS server *via* these metabolic pathways (Nazir et al., 2018). Metabolic pathways of the human host and pathogen were manually compared to find unique and common pathways in the pathogen. KEGG automatic annotation server (KAAS) (Moriya et al., 2007) employs BLASTp analysis for these metabolic pathways. KEGG orthology (KO) identifiers were provided by the KAAS server for proteins (Fatoba et al., 2021). Unique and common pathway proteins were enlisted and categorized into KEGG-dependent and independent proteins. Unique proteins are those involved only in the pathogen-specific pathways but not in humans.

### Druggability analysis of proteins

Screening of essential, non-homologous proteins was further performed by subjecting proteins to BLASTp analysis with a cutoff E-value of 1e-4, against the DrugBank database for identification of druggable proteins (Aslam et al., 2021). All FDA-approved drugs and experimental drugs are contained in the DrugBank database (Law et al., 2014). Druggable targets showed significant matching (80% or more) with FDA-approved drug targets, while proteins not showing any significant matching with already available targets were considered novel targets and used for subsequent analysis.

### Subcellular localization prediction and topology analysis

Proteins are located in different positions, including the cytoplasm, inner membrane, periplasmic membrane, and outer membrane, by subcellular localization prediction. Proteins are categorized as drug or vaccine targets according to their location. Cytoplasmic and outer membrane proteins are considered suitable drug and vaccine targets, respectively (Jaan et al., 2022). PSORTb v3.0 (http://www.psort.org/psortb/) was employed for the prediction of the subcellular location of the resulting target proteins. Further validation of proteins was needed to ensure the location, which was carried out by a server named subcellular localization predictor (CELLO v2.5) (http://cello.life.nctu.edu.tw/) (Yu et al., 2014). The STRING database v10.5 (http://string-db.org) (Szklarczyk et al., 2016) was employed for the analysis of protein–protein interactions by setting parameters at default. The number of interactions was presented by hub proteins showing node degree (K ≥ 5). The transmembrane topology of proteins was predicted by the TMHMM v0.2 server (Krogh et al., 2001). Proteins spanning the lipid membrane are termed transmembrane proteins and are prioritized as potent drug targets (Shah et al., 2021).

### Protein structure modeling and validation

Proteins prioritized in the previous step were employed in SWISS-MODEL for the generation of 3D structures. The server uses homology modeling techniques for the prediction of protein structures when experimental structures are not accessible. Reliable protein templates with expected accuracy were generated (Jaan et al., 2022). A protein verification tool called “ERRAT” was used to verify the quality of the 3D structure of proteins. ERRAT signifies the quality of protein by a parameter called an overall quality factor, where a high value (≥50) specifies good quality for non-bonded atomic interactions (Aslam et al., 2020). The molecular weight and theoretical PI of proteins were determined by the ExPasy server.

The estimation of protein pocket ability to bind small molecules with high affinity is a valuable step in the identification of drug targets. PockDrug, an online server (http://pockdrug.rpbs.univ-paris-diderot.fr.), was used to calculate druggable scores for the prioritized targets. Protein structure information and ligand proximity assessment were used to predict the pocket druggability of proteins by using different estimation methods *via* the PockDrug server. By using pocket estimation methods, consistent druggability results were obtained, which differentiated more druggable proteins from less druggable proteins (Shah et al., 2021).

### Reverse vaccinology: prediction of antigenic proteins

For the evaluation of the antigenic nature of proteins, VaxiJen server v2.0 was employed for outer membrane and extracellular proteins with criteria, i.e., accuracy rate 70–89% and probability score >0.5 (Doytchinova and VaxiJen, 2007). The allergenicity of the shortlisted proteins was evaluated by the AllergenFP server to distinguish allergens from non-allergens. Allergic proteins that are harmful to the host were discarded in this evaluation (Dimitrov et al., 2014). Different parameters of proteins were assessed by the ExPasy ProtParam tool, calculating molecular weight, theoretical PI, instability index, aliphatic index, half-life, number of amino acids, and GRAVY (Grand Average of Hydropathicity) (Qasim et al., 2023).

### Prediction of T-cell epitopes

For a specific pathogen, vaccine targets are identified with the help of T-cell epitopes involved in the elicitation of immune responses. An antigen is processed by an MHC molecule to bind with either cytotoxic or helper T cells (Shah et al., 2021). CD4 or CD8 T cells are stimulated by an epitope defined as the shortest immunogenic peptide having the ability to stimulate immune responses. Two types of major histocompatibility complex (MHC) classes I and II recognized by CD4 and CD8, respectively, are represented by T-cell epitopes (Aslam et al., 2020). MHC prediction was carried out by the Immune Epitope Database (IEDB) server. For the prediction of MHC class-I epitopes, vaccine candidates were subjected to the NetMHCpan 4.1 web server with a percentile rank <0.2, recommended by the IEDB server (Fleri et al., 2017). MHC class-II epitopes were predicted by the IEDB recommendation of 2.22 with an adjusted rank of <0.5. Further evaluation of preferred epitopes was performed.

### Prediction of B-cell epitopes

The solvent-exposed regions in the antigen can be identified by the prediction of B-cell epitopes, by which antibodies can be recognized (Nazir et al., 2018). For the prediction of B-cell epitopes, different servers were used. BCPreds, FBCPreds, AAP, and BepiPred online servers were used to predict linear B-cell epitopes. BCPred uses the SVM method, which has 5-fold cross-validation and utilizes five different kernel methods. Linear-length B-cell epitopes were predicted by subsequent kernel followed by FBCPred. Biochemical properties such as amino acid composition, hydrophobicity, hydrophilicity, secondary structure, and surface accessibility of peptides were used to predict linear epitopes by BepiPred (IEDB) (Solanki and Tiwari, 2018).

### Antigenicity, allergenicity, toxicity, and conservancy analyses

A designated vaccine candidate must be able to act as an antigen. For this purpose, the VaxiJen v2.0 server was employed with a threshold value of >0.4. The sequence alignment method is used in the VaxiJen server, and the physicochemical properties of peptides are used to confirm their antigenicity (Doytchinova and VaxiJen, 2007). The allergenic properties of vaccine candidates were measured by the AllergenFP server. Another server, ToxinPred, was employed to check the toxic and non-toxic peptides, where non-toxic peptides were selected for further evaluation (Jaan et al., 2022). The conservancy level of epitopes was evaluated by the IEDB server by setting parameters at default, where conserved or variable epitopes could be assessed (Jaan et al., 2022). These properties of epitopes were evaluated for the construction of chimeric vaccines.

### Multi-epitope vaccine construction

Multi-epitope vaccine constructs were generated by joining overlapped epitopes of MHC-I, MHC-II, and B cells with the help of amino acid linkers (i.e., EAAAK, GGGS, and KK). To increase the immunogenicity of vaccine constructs, adjuvants were added to the sequence. Four adjuvants, i.e., HBHA protein, L7/L12 ribosomal proteins, beta-defensin, and HBHA conserved sequences, were added to improve the efficacy of vaccine models. PADRE peptide sequences, containing 13 amino acids (i.e., AKFVAAWTLKAAA), were also added, which helped induce CD4+ T cells (Aslam et al., 2020). PADRE sequences help to overcome the problems of polymorphism and elicit better immune responses. Different combinations of vaccine constructs were made and checked for immunogenicity, toxicity, and allergenicity. The SOLPro server was employed to check the solubility of vaccine constructs (Magnan et al., 2009).

### Analysis of physicochemical properties

Intrinsic physical and chemical characteristics of a substance are defined as physicochemical properties. For the estimation of physicochemical properties, the ProtParam tool was used, which calculated the number of amino acids, half-life, theoretical PI, instability index, aliphatic index, molecular weight, and GRAVY of vaccine models (Jaan et al., 2022).

### Structure analysis

For the prediction of secondary structure, two online servers, i.e., PSIPRED and SOPMA, were used. Properties such as transmembrane helices, bend regions, random coils, and beta sheets can be assessed through these self-optimized prediction methods. A graphic presentation of proteins is obtained as output (Jaan et al., 2022). 3D modeling of chimeric constructs was generated by Phyre2, increasing the accuracy of alignment by using the alignment of the Hidden Markov model (Raza et al., 2021). GalaxyRefine and 3DRefine servers were employed for the refinement of predicted 3D models, in which side chains are rebuilt and repacked. The probable errors of 3D models were checked by the ProSA-web server (Jaan et al., 2022). The Ramachandran plot was obtained by an online server called PROCHECK, which explains the stereochemical properties of the protein (Shah et al., 2021). Phi/Psi angles were assessed by the Ramachandran plot for a comprehensive understanding of the protein backbone.

### Disulfide engineering

Vaccine constructs were disulfide-engineered by DbD2, to check the stability and conformational entropy of the protein. Increased stability and decreased conformational entropy of protein can be confirmed by the accessibility of protein, and it can add novel disulfide bonds (Craig and Dombkowski, 2013).

### Molecular docking analysis

An energy-minimizing step used to obtain a stable structural configuration of vaccine targets with the ligands is determined by molecular docking analysis. Immune receptor–peptide interactions were inferred by docking vaccine constructs with six various human HLA alleles using the PatchDock server. More refinement and re-scoring of docked complexes were performed by the Fast Interaction Refinement in Molecular Docking (FireDock) server, which gave the 10 best models as a result. Based on the lowest binding energy, the vaccine construct (V5) was prioritized as the best-docked complex (Jaan et al., 2022). Furthermore, TLR4 (toll-like receptor) acquired from PDB (2Z65) was docked with vaccine construct V5 in the same way by the PatchDock server. The top 10 generated models from the FireDock server were redirected to PatchDock for refinement. Assumed refined solutions were recognized by the binding score and global binding energies of docked refined models.

### Molecular dynamic simulation

The stability of the docked complex and molecules' behavior was assessed by the molecular dynamic approach. The interaction between the designed vaccine and receptor was estimated by the iMOD server. Four main factors are measured by this tool by estimating the direction and range of basic movements of the docked complex. These factors include Eigenvalue, B-factors, covariance, and deformability. A high Eigenvalue indicates the value of much harder deformation (Jaan et al., 2022).

### *In silico* expression analysis and immune simulation

The possible expression level of vaccine constructs was tested by using the Java Codon Adaptation tool (JCAT), which used back-translated amino acid sequences. *E. coli* (K12 strain) was selected for the expression of the protein. The high expression level in *E. coli* was verified by the consequential GC content and codon adaptation index (CAI) (Jaan et al., 2022). The rho-independent transcription terminators, cleavage sites of some restriction enzymes, and prokaryotic ribosome binding sites were dodged. SnapGene software was used to introduce codon-adapted sequences into plasmid vector pET30a (+), to construct recombinant plasmid sequences (Solanki and Tiwari, 2018). C-ImmSim server was employed for immune simulation to predict the production of interferons, antibodies, and cytokines in response to a foreign particle. Parameters were set to default (Puzone et al., 2002).

## Results

### Core protein retrieval and CD-HIT analysis

The core genome method uses conserved sequences of available complete genome strains in accordance with the reference genome. A total of 1,415 core proteins were retrieved from 14 complete genome strains of *P. melaninogenica*. Some proteins are produced in response to duplication events taking place in organisms within one species, known as paralogous proteins. To reduce redundancy in sequences, proteins were subjected to CD-HIT analysis (Li and Godzik, 2006). A total of 1,414 non-paralogous proteins were obtained by subtracting one duplicate protein from the core protein sequences. The threshold was set to 0.6 (60%) sequence similarity.

### Human host non-homolog analysis

For the identification of novel targets, bacterial proteins were subjected to homology filters as an essential step for the screening of human host homologs. After removing the proteins homologous to the host proteins, 1,295 candidates were obtained by screening against the human proteome database. Different metabolic reactions such as homeostasis, development, defense system, and physiological functions are performed by the gut flora, which colonizes the human body throughout life (Kho and Lal, 2018). The scanning of core proteins against the human gut microbiota was performed by standalone BLASTp, to prevent the likelihood of serious complications in the host. The resulting 615 proteins were used for downstream analysis.

### Identification of essential, virulent, antibiotic-resistant, and interacting proteins

A minimal set of genes required to perform basic cellular functions and important for the survival of an organism are defined as essential genes. A database of essential genes (DEG) based on BLASTp scanning was employed to dig out essential proteins of the pathogen. As a result, 123 proteins were recognized as essential for the maintenance of cellular functions in *P. melaninogenica*. The virulence factor database (VFDB) was used to identify the virulence proteins of the pathogen. The pathogenic capacity of bacteria to infect the host is increased by virulence factors (Aslam et al., 2020). By subjecting proteins to BLASTp scanning, VFDB calculated 19 proteins as virulence factors.

Antibiotic resistance proteins were determined by Antibiotic-Resistant Gene ANNOTation (ARG-ANNOT) (Gupta et al., 2014). This analysis determined three proteins as resistant to antibiotics, which might act as important drug targets in future studies. Host–pathogen protein–protein interactions (HP-PPI) mediate infectious diseases in response to molecular cross-conferences between host and pathogens. For treating infectious diseases, such proteins need to be identified for the discovery of potential drug targets. Proteins were subjected to BLASTp against HPIDB v2.0, which identified only one interacting protein (Ammari et al., 2016). The proteins from this analysis have the potential to be selected as alternative drug targets and deserve further experimental investigation.

### Identification of pathogen-specific metabolic pathways

Metabolic pathway analysis is used to find out the preferable drug target as involved in pathogen-specific unique pathways (Dorella et al., 2013). The KEGG database was used to predict the metabolic pathways of *Homo sapiens* (humans) and *P. melaninogenica*, which were 345 and 94, respectively. A manual comparison of human and bacterial metabolic pathways showed that 25 unique pathways were present but absent in the human host, while the remaining 69 pathways were common to both humans and bacteria. The KAAS server was employed for the functional pathway analysis of 114 essential, non-homologous pathogen proteins *via* BLASTp, which assigned them a KO (KEGG orthology) identifier (Fatoba et al., 2021). Out of 114 proteins, only 5 were involved in pathogen-specific unique pathways called KEGG-dependent (Table 1), while the rest of the 109 proteins were categorized as KEGG-independent.

**Table 1:** *P. melaninogenica* unique pathway proteins.

**Sr. no**.	**Protein IDs**	**KO identifiers**	**Pathway IDs**
1	gi|302149751|gb|ADK96013.1|	K02517	Lipopolysaccharide biosynthesis
2	gi|302150676|gb|ADK96937.1|	K05515	O-Antigen repeat unit biosynthesis
3	gi|302150062|gb|ADK96324.1|	K00865	Biosynthesis of secondary metabolites
4	gi|302149000|gb|ADK95262.1|	K02551	Biosynthesis of secondary metabolites
5	gi|302149929|gb|ADK96191.1|	K03787	Biosynthesis of secondary metabolites
6	gi|302150384|gb|ADK96645.1|	K00919	Biosynthesis of secondary metabolites
7	gi|302150673|gb|ADK96934.1|	K11753	Biosynthesis of secondary metabolites
8	gi|302150062|gb|ADK96324.1|	K00865	Microbial metabolism in diverse environments
9	gi|302149329|gb|ADK95591.1|	K06881	Microbial metabolism in diverse environments
10	gi|302149556|gb|ADK95818.1|	K08678	Biosynthesis of nucleotide sugars
11	gi|302150676|gb|ADK96937.1|	K05515	Beta-Lactam resistance
12	gi|302151208|gb|ADK97469.1|	K01448	Cationic antimicrobial peptide (CAMP) resistance
13	gi|302150566|gb|ADK96827.1|	K07165	Two-component system
14	gi|302150409|gb|ADK96670.1|	K03217	Quorum sensing
15	gi|302150820|gb|ADK97081.1|	K03086	Flagellar assembly
16	gi|302150409|gb|ADK96670.1|	K03217	Bacterial secretion system

The potential therapeutic targets were represented by the metabolic pathway analysis of non-homologous, essential human proteins. Suitable and least-targeted were identified by applying filters to reduce time, labor, and resources. Druggability screening of the prioritized proteins was performed to find novel druggable targets, which demonstrated that in KEGG-dependent, one protein is a novel target, while in KEGG-independent proteins, 90 proteins are novel and have the potential to be explored as potential drug targets.

### Prediction of subcellular localization

Suitable and effective targets can be identified by predicting subcellular location for a comprehensive understanding of the function and mechanisms of proteins. Purification and assay of membrane-localized proteins are critical, so cytoplasmic proteins are favored as potent drug targets (Mondal et al., 2015). For suitable vaccine targets, outer membrane and extracellular proteins were prioritized for elicitation of better immune responses as membrane proteins were exposed to host cells (Nogueira et al., 2021). Subcellular location prediction by CELLO and PSORTb tools showed three proteins to be cytoplasmic and only one protein to be outer membrane in the case of KEGG-dependent proteins. In the case of KEGG-independent proteins, 49 proteins were found to be cytoplasmic and 23 proteins to be outer membrane in location. These proteins were prioritized as drug or vaccine targets for further analysis in accordance with their location.

### Drug target prioritization

**Figure 2:**
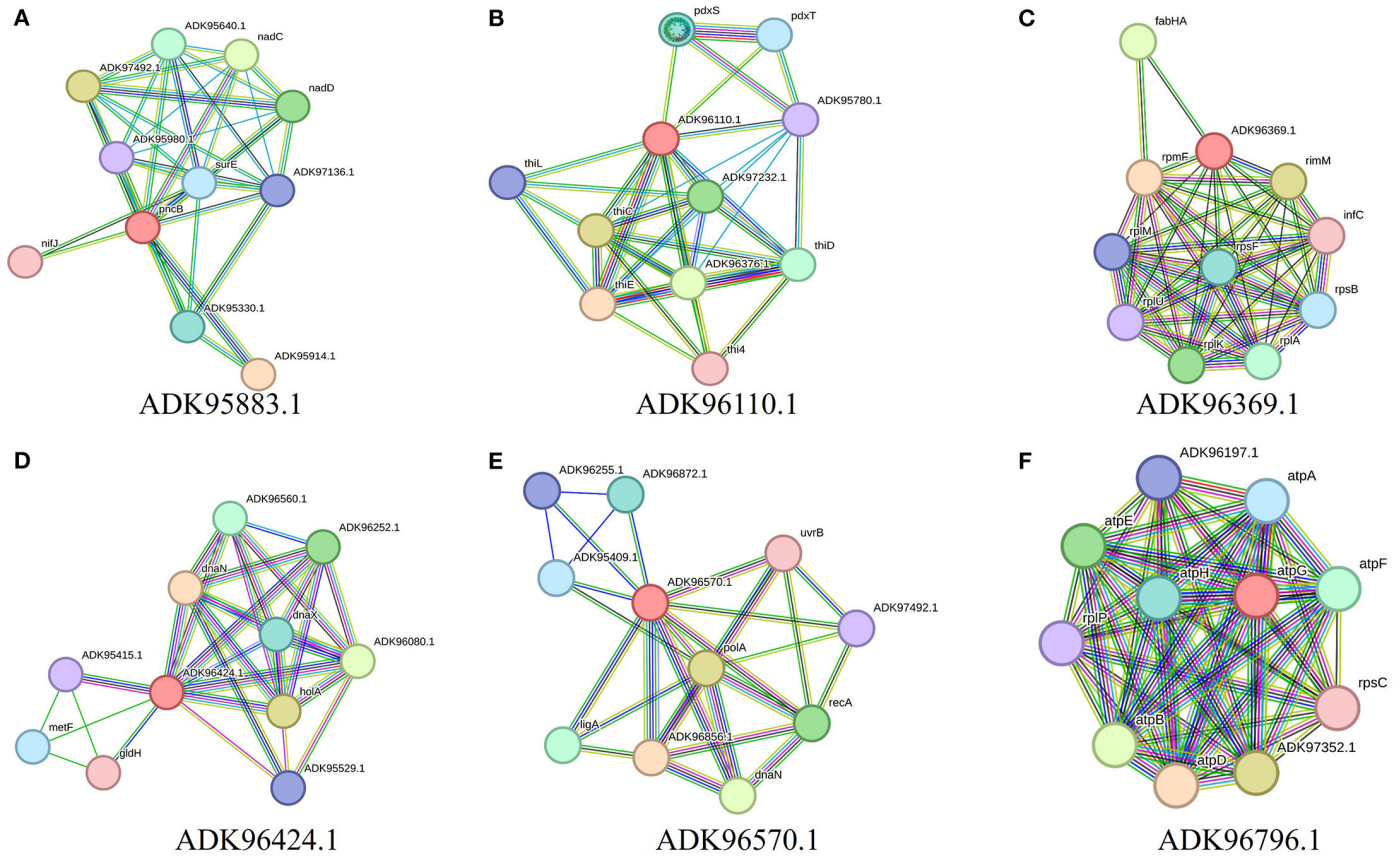
Interaction analysis of predicted drug targets with other proteins using STRING database where query proteins are indicated by red color. The proteins with the best predicted three-dimensional structures: **(A)** ADK95883.1, **(B)** ADK96110.1, **(C)** ADK96369.1, **(D)** ADK96424.1, **(E)** ADK96570.1, and **(F)** ADK96796.1, are shown to summarize the drug target's PPI list.

Further characterization of selected targets was performed by STRING v10.5 databases for the estimation of protein–protein interactions. A significant number of interactions were represented by node degree (K) ≥5 as hub proteins (Szklarczyk et al., 2016). This characterization prioritized 20 proteins as suitable targets (Figure 2). The TMHMM server was employed for the prediction of transmembrane helices set to be less than or equal to 1. Due to multiple transmembrane helices, there may be complications in the cloning, expression, and purification of proteins (Solanki and Tiwari, 2018). For this purpose, proteins with more than one helix were excluded, and 18 proteins were selected (Table 2). The molecular weight of selected targets was calculated by the ExPasy tool, which prioritized proteins with < 110 KDa molecular weight. Proteins submitted to SWISS-MODEL were checked for model quality estimation by different parameters, i.e., percentage identity, Q-mean, and GMQE scores, which prioritized three drug targets. Protein pocket ability prediction is one of the crucial steps in the development of therapeutic drugs to ensure the affinity of proteins with small drug-like molecules. The PockDrug server was employed for this purpose, which identified significant druggable targets with a drug score of >0.5 (Borrel et al., 2015). Furthermore, the ERRAT server was used to evaluate the quality of models, which was considered significant (85%) for all models.

**Table 2:** Analysis of shortlisted druggable proteins to check transmembrane alpha helices, molecular weight, and node degree (STRING analysis).

**Sr. no**.	**Protein ID**	**Protein name**	**TMHMM No**.	**M. wt (KDa)**	**Query length**	**STRING**
**Metabolic pathway-dependent protein**						
1	ADK97401.1	Dihydroneopterin aldolase	0	13	23	6.55
**Metabolic pathway-independent proteins**						
1	ADK95262.1	2-succinyl-5-enolpyruvyl-6-hydroxy-3-cyclohexene-1-carboxylic-acid synthase	0	61	113	6
2	ADK95358.1	Pyridoxal 5′-phosphate synthase	0	31	77	5.09
3	ADK95511.1	Hypothetical protein	0	23	35	6.36
4	ADK95702.1	NAD-dependent DNA ligase OB-fold domain protein	0	74	155	6.55
5	ADK95883.1	Nicotinate phosphoribosyltransferase	0	49	80	7.82
6	ADK95886.1	Glycosyltransferase	0	33	49	6
7	ADK96013.1	Lipid A biosynthesis (KDO)2-(lauroyl)-lipid IVA acyltransferase	1	39	46	6.55
8	ADK96033.1	YbbR-like protein	0	33	46	6.36
9	ADK96110.1	Putative phosphomethylpyrimidine kinase	0	36	73	7.09
10	ADK96369.1	Putative ACR, COG1399	0	19	34	8.55
11	ADK96424.1	Hypothetical protein	0	43	74	7.45
12	ADK96445.1	Exodeoxyribonuclease VII, large subunit	0	48	89	5.82
13	ADK96570.1	UvrD/REP helicase	0	103	162	6.73
14	ADK96645.1	4-(cytidine 5′-diphospho)-2-C-methyl-D-erythritol kinase	0	31	66	6.18
15	ADK96796.1	ATP synthase F1, gamma subunit	0	36	64	8.73
16	ADK96878.1	Hypothetical protein	1	18	23	6.36
17	ADK96880.1	Hydrolase, tatd family	0	30	56	5.64

### Antigenic membrane protein selection

Surface-exposed or outer membrane proteins which were exposed to host cells were prioritized for the reverse vaccinology, in the case of gram-negative bacteria. Certain characteristics (i.e., instability index, aliphatic index, PI, GRAVY, and molecular weight) of 24 outer membrane proteins were analyzed by the ProtParam tool. The AlgPred server was employed to check the allergenicity of proteins by using the hybrid method for the best vaccine candidate selection. Antigenicity was estimated by the VaxiJen server based on the threshold of >0.5. Two proteins with IDs ADK95685.1 and ADK97014.1 were selected as the best vaccine targets used for subsequent analysis. By this analysis, it was ensured that induced immune responses were aimed to target the pathogen, not the host cells.

### Prediction of lead epitopes

Targeted immune responses are induced by the short peptide fragments of a peptide vaccine; accordingly, allergenic sequences are avoided. A variety of peptides termed epitopes can bind to MHC molecules with high affinity as mutations are possible in MHC-binding epitopes caused by pathogens (Shah et al., 2021). One of the major keystones in the development of vaccines is the B- and T-cell epitope prediction (Li et al., 2014). MHC-I and MHC-II molecules provide both types of T cells, i.e., (i) cytotoxic T cells and (ii) helper T cells, with epitopes. High-binding affinity epitopes were predicted by multiple prediction sources to find potent vaccine-candidate epitopes. For the prediction of MHC-binding epitopes in two prioritized vaccine candidates, an IEDB server was employed. For MHC-I binding, the NetMHCpan 4.1 server was used with criteria of <0.2 percentile rank. Hydrogen binding can attach peptides with only 8 to 10 amino acids due to the closed binding site cleft of MHC-I molecules (Nazir et al., 2018). Criteria for binding with human alleles were set to 9-mer length, and predicted 19 and 16 epitopes for both vaccine candidates were used for further analysis. These epitopes were further filtered by toxicity, antigenicity, conservancy, and immunogenicity analyses, which resulted in 5 and 6 final epitopes for chimera vaccine construction.

Due to open and shallow pockets, prediction by MHC-II molecules is less accurate. The IEDB-recommended 2.22 method was employed for the prediction of MHC-II epitopes with a percentile rank <0.5, and the 15-mer length of amino acids was selected due to the higher binding affinity of these molecules as compared with MHC-I (Shah et al., 2021). After screening, the final MHC-II and MHC-I epitopes were selected (Supplementary Tables S1, S2). For the prediction of linear B-cell epitopes, different online servers, i.e., BCPred, BepiPred, FBCPred, and AAP, were employed. The predicted epitopes of T and B cells were compared for the determination of overlapping epitopes (Table 3). These overlapping epitopes may be useful in the development of a vaccine against *P. melaninogenica* in future. Conformational B-cell epitopes have an important role in antigen and antibody association, but unfortunately, these cannot be predicted due to the inaccessibility of the 3D structures of all the proteins.

**Table 3:** Comparative analysis of predicted epitopes (bold highlights the selected epitopes).

**Protein IDs**	**Sr. no**.	**Final positions**	**Final B-cell epitopes**
ADK95685.1	1	2, −50	ATRTINGREYTKVDFENKLRHLSDAQELAAMLNELKLPWYYSDFRGKQL
	**2**	**55–83**	DTNNVQRRCKTFRLRKKHGGYREITAPKG
	**3**	**100–127**	DEPTPWAFGFVCGRSVVDNARPHVGKRY
	4	168–199	ATVRTKNNKEVLAQGFATSPTLSNFICREMDK
	**5**	**208–254**	QGITFTRYADDLTFSSDTDILRPQGELVQQVKAIVERYGFRLNEEKT
	**6**	**268–294**	LMVTEKVNVSRRYVREIRSLLYIWERY
	7	303–325	AWKSYRQQHGKTKGHQHCVPLNA
	8	349–375	VSRYTSLQQRSKGDIKEVAYKAYMGKY
	9	377–417	SNSTEDRMTSANVLPNDNTSSRQLGATSSNPYDPRKKSKRI
ADK97014.1	**1**	**15–45**	VQVSAQSGTNSPYSQYGLGALASQATSFNRG
	2	52–75	GFHERNQVNYANPASYASVDSLSF
	**3**	**82–109**	SLQLTNFEENGNKVNAKNADIEYVVASF
	4	121–161	LLPYTNVGYNFSNTQNVNAFPSTSSVNATYSNAYNGSGGLH
	**5**	**165–236**	LGAGWEPFKGFSFGANIGYLWGTLNRNSTNTYSDSYVNTLSKNYSAQVKSYKVDFGAQYTYAVDKKNELTLG
	6	253–274	LISTNSQTSISDTTRYVVSNSL
	**7**	**278–329**	HTFGVGLMWNHNNRLKFGVDYQLQKWAKLKYPQLTTVNGTTSYNLVDGQFND
	**8**	**332–379**	KFTLGGDYCKGERYRGFFSRMHYRAGFSYASPYLKINGVDGPRELSAS
	**9**	**390–427**	YNNRSMLNISAEWVNQSVTGMIKENMFRINVGFTFNER

### Vaccine construct modeling

For the construction of chimera vaccine constructs, different combinations of non-toxic, antigenic, and conserved lead epitopes were chosen. On the N-terminal of prioritized epitopes, adjuvants were added to enhance the immunogenic nature of the multi-epitope vaccine. Linkers, i.e., EAAAK, GGGS, and KK, were used to join the adjuvants. Allergenicity and antigenicity of the constructs affected by adjuvants were analyzed by the different combinations of adjuvants (Solanki and Tiwari, 2018). The conformation of designed constructs was not altered by adding linkers. Around the global population, the problems triggered by polymorphisms in HLA-DR molecules can be overcome by adding PADRE sequences (synthetic peptides containing 13 amino acids) to vaccine constructs. Better immune protection and cytotoxic T-lymphocyte (CTL) responses were provided by PADRE sequences (Wu et al., 2010). Different combinations of lead epitopes, adjuvants, and PADRE sequences provided eight vaccine constructs, i.e., V1, V2, V3, V4, V5, V6, V7, and V8 (Table 4).

**Table 4:** Designed multi-epitope vaccine sequences.

**Sr. no**.	**Epitope sequence position**	**Complete sequence of vaccine construct**
1	ADK95685.1 (100–127, 208–254), and ADK97014.1 (52–75, 121–161, 253–274, 332–379, 390–427)	EAAAKMSDLKNLAETLVNLTVKDVNELAAILKDEYGIEPAAAAVVMAGPGAEAAEEKTEFDVILKSAGASKLAVVKLVKD LTGAGLKEAKDMVDGAPAAIKSGISKDEAEALKKQLEEAGAEVELKEAAAKAKFVAAWTLKAAAGGGSDEPTPWAFGFVCGR SVVDNARPHVGKRYGGGSQGITFTRYADDLTFSSDTDILRPQGELVQQVKAIVERYGFRLNEEKTGGGSAKFVAAWTLKAAA GGGSGFHERNQVNYANPASYASVDSLSFHEYGAEALERAGLLPYTNVGYNFSNTQNVNAFPSTSSVNATYSNAYNGSGGLHH EYGAEALERAGLISTNSQTSISDTTRYVVSNSLHEYGAEALERAGKFTLGGDYCKGERYRGFFSRMHYRAGFSYASPYLKIN GVDGPRELSASHEYGAEALERAGYNNRSMLNISAEWVNQSVTGMIKENMFRINVGFTFNERHEYGAEALERAGAKFVAAWTL KAAAGGGS
2	ADK95685.1 (100–127, 208–254), and ADK97014.1 (52–75, 121–161, 253–274, 332–379, 390–427)	EAAAKMAENPNIDDLPAPLLAALGAADLALATVNDLIANLRERAEETRAETRTRVEERRARLTKFQEDLPEQFIELRDK FTTEELRKAAEGYLEAATNRYNELVERGEAALQRLRSQTAFEDASARAEGYVDQAVELTQEALGTVASQTRAVGERAAKL VGIELEAAAKAKFVAAWTLKAAAGGGSDEPTPWAFGFVCGRSVVDNARPHVGKRYGGGSQGITFTRYADDLTFSSDTDIL RPQGELVQQVKAIVERYGFRLNEEKTGGGSAKFVAAWTLKAAAGGGSGFHERNQVNYANPASYASVDSLSFHEYGAEALE RAGLLPYTNVGYNFSNTQNVNAFPSTSSVNATYSNAYNGSGGLHHEYGAEALERAGLISTNSQTSISDTTRYVVSNSLHE YGAEALERAGKFTLGGDYCKGERYRGFFSRMHYRAGFSYASPYLKINGVDGPRELSASHEYGAEALERAGYNNRSMLNIS AEWVNQSVTGMIKENMFRINVGFTFNERHEYGAEALERAGAKFVAAWTLKAAAGGGS
3	ADK95685.1 (100–127, 208–254), and ADK97014.1 (52–75, 121–161, 253–274, 332–379, 390–427)	EAAAKMAENSNIDDIKAPLLAALGAADLALATVNELITNLRERAEETRRSRVEESRARLTKLQEDLPEQLTELREKFTA EELRKAAEGYLEAATSELVERGEAALERLRSQQSFEEVSARAEGYVDQAVELTQEALGTVASQVEGRAAKLVGIELEAAA KAKFVAAWTLKAAAGGGSDEPTPWAFGFVCGRSVVDNARPHVGKRYGGGSQGITFTRYADDLTFSSDTDILRPQGELVQQ VKAIVERYGFRLNEEKTGGGSAKFVAAWTLKAAAGGGSGFHERNQVNYANPASYASVDSLSFHEYGAEALERAGLLPYTN VGYNFSNTQNVNAFPSTSSVNATYSNAYNGSGGLHHEYGAEALERAGLISTNSQTSISDTTRYVVSNSLHEYGAEALERA GKFTLGGDYCKGERYRGFFSRMHYRAGFSYASPYLKINGVDGPRELSASHEYGAEALERAGYNNRSMLNISAEWVNQSVT GMIKENMFRINVGFTFNERHEYGAEALERAGAKFVAAWTLKAAAGGGS
4	ADK95685.1 (100–127, 208–254), and ADK97014.1 (52–75, 121–161, 253–274, 332–379, 390–427)	EAAAKGIINTLQKYYCRVRGGRCAVLSCLPKEEQIGKCSTRGRKCCRRKKEAAAKAKFVAAWTLKAAAGGGSDEPTPWA FGFVCGRSVVDNARPHVGKRYGGGSQGITFTRYADDLTFSSDTDILRPQGELVQQVKAIVERYGFRLNEEKTGGGSAKFVA AWTLKAAAGGGSGFHERNQVNYANPASYASVDSLSFHEYGAEALERAGLLPYTNVGYNFSNTQNVNAFPSTSSVNATYSNA YNGSGGLHHEYGAEALERAGLISTNSQTSISDTTRYVVSNSLHEYGAEALERAGKFTLGGDYCKGERYRGFFSRMHYRAGF SYASPYLKINGVDGPRELSASHEYGAEALERAGYNNRSMLNISAEWVNQSVTGMIKENMFRINVGFTFNERHEYGAEALER AGAKFVAAWTLKAAAGGGS
5	ADK95685.1 (55–83, 268–294), and ADK97014.1 (15–45, 82–109, 165–236, 278–329)	EAAAKMSDLKNLAETLVNLTVKDVNELAAILKDEYGIEPAAAAVVMAGPGAEAAEEKTEFDVILKSAGASKLAVVKLVK DLTGAGLKEAKDMVDGAPAAIKSGISKDEAEALKKQLEEAGAEVELKEAAAKAKFVAAWTLKAAAGGGSDTNNVQRRCKTF RLRKKHGGYREITAPKGGGGSLMVTEKVNVSRRYVREIRSLLYIWERYGGGSAKFVAAWTLKAAAGGGSVQVSAQSGTNSP YSQYGLGALASQATSFNRGHEYGAEALERAGSLQLTNFEENGNKVNAKNADIEYVVASFHEYGAEALERAGLGAGWEPFKG FSFGANIGYLWGTLNRNSTNTYSDSYVNTLSKNYSAQVKSYKVDFGAQYTYAVDKKNELTLGHEYGAEALERAGHTFGVGL MWNHNNRLKFGVDYQLQKWAKLKYPQLTTVNGTTSYNLVDGQFNDHEYGAEALERAGAKFVAAWTLKAAAGGGS
6	ADK95685.1 (55–83, 268–294), and ADK97014.1 (15–45, 82–109, 165–236, 278–329)	EAAAKMAENPNIDDLPAPLLAALGAADLALATVNDLIANLRERAEETRAETRTRVEERRARLTKFQEDLPEQFIELRDK FTTEELRKAAEGYLEAATNRYNELVERGEAALQRLRSQTAFEDASARAEGYVDQAVELTQEALGTVASQTRAVGERAAKLV GIELEAAAKAKFVAAWTLKAAAGGGSDTNNVQRRCKTFRLRKKHGGYREITAPKGGGGSLMVTEKVNVSRRYVREIRSLLY IWERYGGGSAKFVAAWTLKAAAGGGSVQVSAQSGTNSPYSQYGLGALASQATSFNRGHEYGAEALERAGSLQLTNFEENGN KVNAKNADIEYVVASFHEYGAEALERAGLGAGWEPFKGFSFGANIGYLWGTLNRNSTNTYSDSYVNTLSKNYSAQVKSYKV DFGAQYTYAVDKKNELTLGHEYGAEALERAGHTFGVGLMWNHNNRLKFGVDYQLQKWAKLKYPQLTTVNGTTSYNLVDGQF NDHEYGAEALERAGAKFVAAWTLKAAAGGGS
7	ADK95685.1 (55–83, 268–294), and ADK97014.1 (15–45, 82–109, 165–236, 278–329)	EAAAKMAENSNIDDIKAPLLAALGAADLALATVNELITNLRERAEETRRSRVEESRARLTKLQEDLPEQLTELREKFTA EELRKAAEGYLEAATSELVERGEAALERLRSQQSFEEVSARAEGYVDQAVELTQEALGTVASQVEGRAAKLVGIELEAAAK AKFVAAWTLKAAAGGGSDTNNVQRRCKTFRLRKKHGGYREITAPKGGGGSLMVTEKVNVSRRYVREIRSLLYIWERYGGGS AKFVAAWTLKAAAGGGSVQVSAQSGTNSPYSQYGLGALASQATSFNRGHEYGAEALERAGSLQLTNFEENGNKVNAKNADI EYVVASFHEYGAEALERAGLGAGWEPFKGFSFGANIGYLWGTLNRNSTNTYSDSYVNTLSKNYSAQVKSYKVDFGAQYTYA VDKKNELTLGHEYGAEALERAGHTFGVGLMWNHNNRLKFGVDYQLQKWAKLKYPQLTTVNGTTSYNLVDGQFNDHEYGAEA LERAGAKFVAAWTLKAAAGGGS
8	ADK95685.1 (55–83, 268–294), and ADK97014.1 (15–45, 82-109, 165–236, 278–329)	EAAAKGIINTLQKYYCRVRGGRCAVLSCLPKEEQIGKCSTRGRKCCRRKKEAAAKAKFVAAWTLKAAAGGGSDTNNVQR RCKTFRLRKKHGGYREITAPKGGGGSLMVTEKVNVSRRYVREIRSLLYIWERYGGGSAKFVAAWTLKAAAGGGSVQVSAQS GTNSPYSQYGLGALASQATSFNRGHEYGAEALERAGSLQLTNFEENGNKVNAKNADIEYVVASFHEYGAEALERAGLGAGW EPFKGFSFGANIGYLWGTLNRNSTNTYSDSYVNTLSKNYSAQVKSYKVDFGAQYTYAVDKKNELTLGHEYGAEALERAGHT FGVGLMWNHNNRLKFGVDYQLQKWAKLKYPQLTTVNGTTSYNLVDGQFNDHEYGAEALERAGAKFVAAWTLKAAAGGGS

### Antigenicity, allergenicity, and solubility analysis of vaccine constructs

The antigenic behavior of vaccine constructs was examined by the AntigenPro server with a cutoff value of >0.90. The VaxiJen server was employed to estimate the antigenicity, with a threshold of 0.75 showing good antigenic behavior. The allergic nature of constructs was checked by the AllergenFp, and allergic constructs were excluded as being harmful to the human host. The SOLPro server with a probability score of >0.5 was employed to analyze the solubility of the designed chimeric constructs. GC content (30–70%) and CAI value (0.90–1.0) were considered significant as inferred from the results. The ProtParam tool was used to evaluate properties such as the number of amino acids, aliphatic index, instability index, PI, molecular weight, and GRAVY. Eventually, vaccine constructs V5 and V8 fulfilled the standard criteria and were selected for further investigations (Supplementary Tables S3, S4).

### Structure prediction of vaccine constructs

A 2D diagram of the vaccine represents the residues involved in the formation of coils, b-sheets, and a-helices. These particular secondary structures of vaccine are involved in its overall structural stability. The 2D diagram indicates a big picture of the complete structure of the vaccine construct. Therefore, prioritized vaccine constructs (V5 and V8) were subjected to PSIPRED and SOPMA servers for the prediction of secondary structure to evaluate the stability of peptides (Raza et al., 2021). The results revealed by servers calculated the percentage of α-helix (36.90), β-strands (10.69), extended strands (21.80), and random coils (30.61) for V5 (Supplementary Figure S1A). Similarly, percentages for V8 were 29.43, 24.44, 11.22, and 34.91, respectively (Supplementary Figure S1B). The function and stability of proteins are critically analyzed by tertiary structure prediction. The Phyre2 server was employed for the prediction of the tertiary structure of prioritized vaccine constructs (V5 and V8). The vaccine construct V8 was deselected due to limited data for three-dimensional structure formation. Hence, V5 was used for tertiary structure, and distortions in protein structure were overcome by refinement of structure. Closeness to the native structure of proteins was acquired by the refinement of models by GalaxyRefine and 3DRefine. Efficient protein structure refinement is obtained by applying knowledge-based force fields and composite physics. Energy minimization steps and repetitive optimization of hydrogen bonding at the atomic level are employed by these online servers for refinement (Raza et al., 2021).

**Figure 3:**
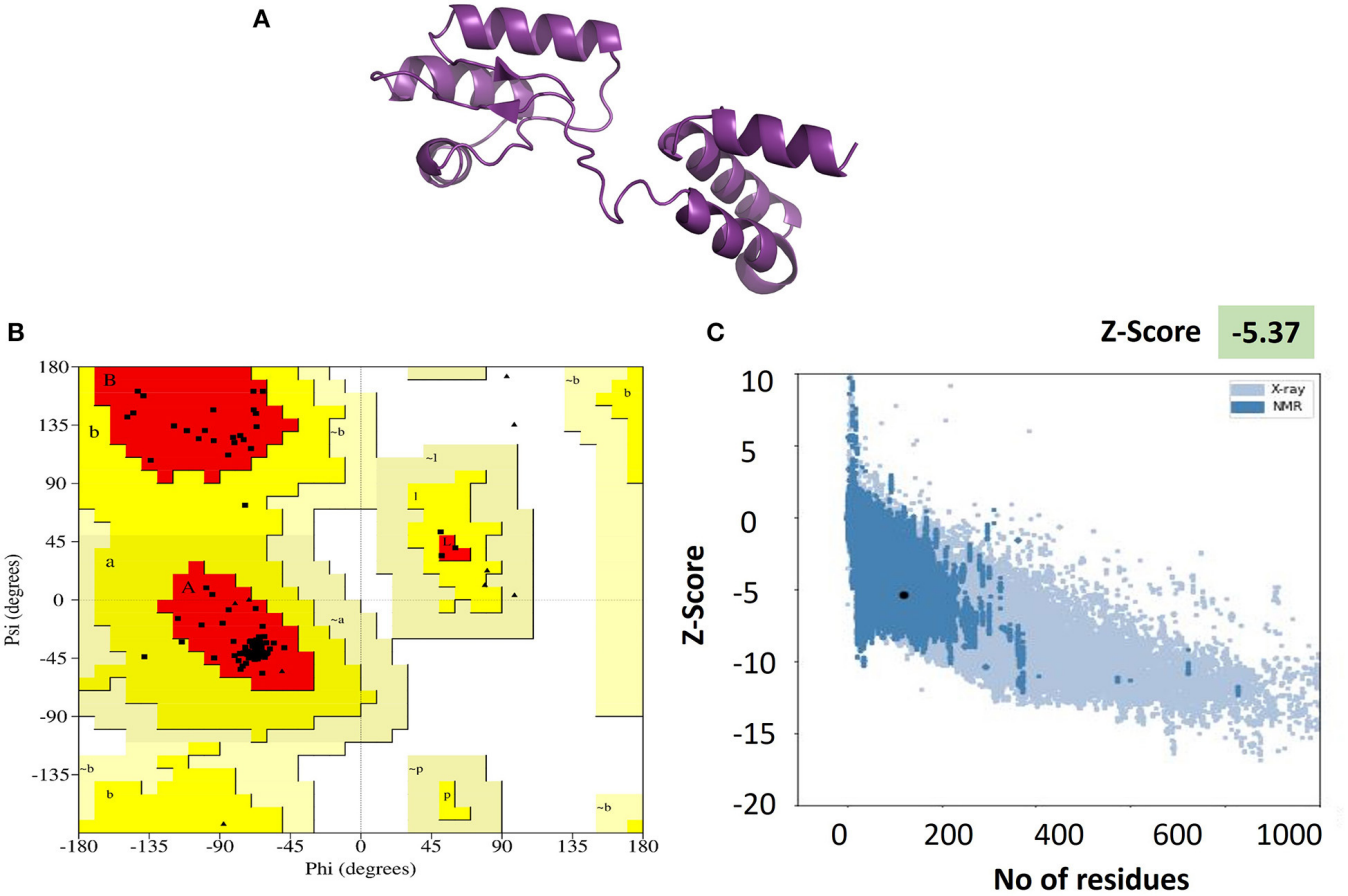
**(A)** Tertiary structure of the vaccine V5 generated and refined by Phyre 2 and GalaxRefine servers, respectively. **(B)** Ramachandran plot exhibiting more than 95% in the Rama-favored regions. **(C)** Vaccine 3D structure validation by ProSA-web. The Z-score of the refined model is −5.37, which is lying inside the score range.

Validation of the refined 3D model was executed by PROCHECK and PROSAweb, which were used to generate the Ramachandran plot. This plot assessment elaborates on the combination and orientation of dihedral angles falling in the disallowed region on the basis of steric hindrance (Maxwell and Popelier, 2017). The Z-score (assessed by PROSA Web) improved from −2.98 to −5.37, which ensured the better quality of models. More than 90% of residues in the most favored regions of the Ramachandran plot indicate the better folding of the protein structure and validate its quality. The results of the Ramachandran plot analysis of our vaccine showed 95.2% of residues in the most favored regions, which indicated the good quality of our vaccine. Visualization of the tertiary structures was made with Pymol v2.5.5 software for better representation (Figure 3).

### Disulfide engineering and molecular docking analysis

The designed vaccine construct was stabilized by performing disulfide engineering, where 13 pairs of amino acids were found to be able to make disulfide bonds predicted by the DbD2 server. However, considering the parameters such as energy and chi3, five pairs were considered favorable. The value of energy was calculated to be <4 kcal/mole for 5 pairs, with a chi3 value between −105 and +109.

Favorable interactions between vaccine constructs and HLA alleles were predicted by molecular docking analysis, an energy minimization step. The vaccine construct (V5) was docked with six different HLA alleles, i.e., 1A6A, 1AQD, 2Q6W, 3C5J, 4MD4, and 5NI9, acquired from PDB by using the PatchDock server. For refinement of models, the results were subjected to the FireDock server. Docked complex V5-1AQD fulfilled the criteria by showing the highest docking score (14814) as compared with the docking score of other docked complexes. Vigorous immune-stimulatory and CTL response effects can be elicited by interactions between TLRs (Rana and Akhter, 2016). The vaccine construct (V5) was further docked with TLR4 (toll-like receptor) to enhance immune responses (Table 5). Profound interactions of the docked complex V5-TLR4 were confirmed through binding energy.

**Table 5:** Docking analysis for the interaction studies between vaccine construct V5 and major immune receptors.

**Sr. no**.	**HLA alleles PDB ID**	**Score**	**Area**	**Hydrogen bond energy**	**Global energy**	**ACE**
1	2Q6W	13,898	1,886.3	−1.25	−15.29	7.27
2	1A6A	14,024	1,894.2	−0.9	−11.84	−2.64
3	5NI9	14,416	1,935.3	−3.07	−16.12	9.23
4	TLR4	14,506	1,932	−2.05	−12.82	13.86
5	4MD4	14,544	1,912.2	−5.03	−21.8	9.32
6	1AQD	14,814	1,867.8	−5.34	−25.38	8.99

### Molecular dynamic simulation

**Figure 4:**
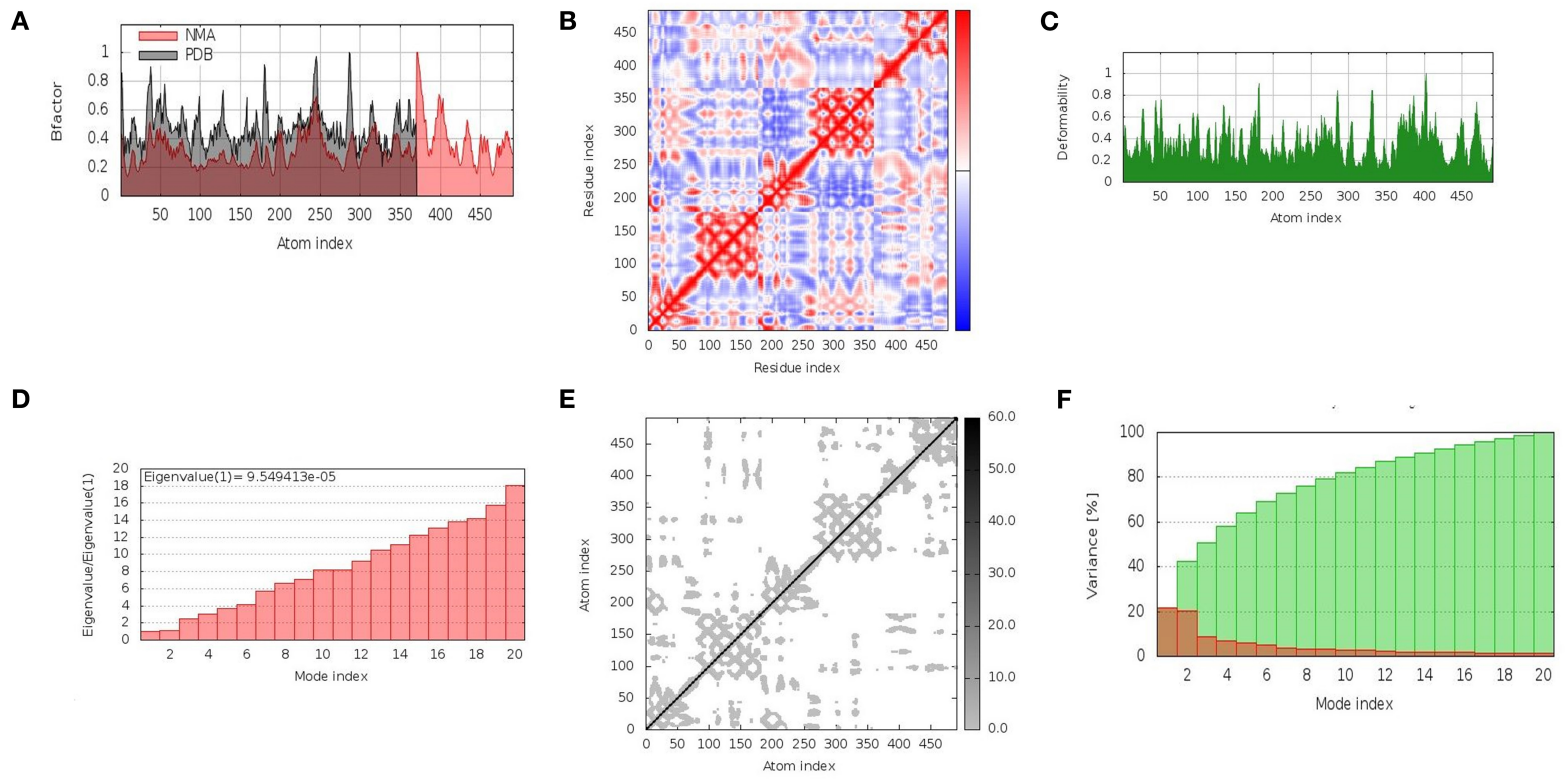
Molecular dynamics simulation of vaccine construct (V5)–TLR4 complex. The stability of the protein–protein complex was examined by **(A)** B-factor, **(B)** covariance matrix of residue index, **(C)** deformability, **(D)** Eigenvalue, **(E)** elastic network analysis, and **(F)** variance.

In the cellular environment, the stability of docked complexes was inferred by molecular dynamic simulation studies. The iMOD server was employed to explore the movement of molecules and atoms. The relation of the docked complex between the NMA and the PDB sector was clearly visualized by the B-factor graph (Figure 4A). The coupling between residues was indicated by a covariance matrix, where the motions, i.e., correlated, uncorrelated, and anti-correlated, of different pairs were indicated by red, blue, and white colors, respectively (Figure 4B). The distortion of each residue was counted as the deformability of the complex, as shown in the graphs obtained by the server with higher peaks indicating the greater deformability (Figure 4C). A low Eigenvalue indicates that the complex can be deformed easily. The motion stiffness of the complex was represented by an Eigenvalue (9.549413e-05) (Figure 4D). The Eigenvalue and variance related to each normal mode were found to be in an inverse relation (Figure 4F). The elastic network model indicated the stiffness of the residues of the vaccine in the form of springs, with darker grays indicating stiffer springs (Figure 4E). The interpretation obtained from the results confirmed the stability of the docked complex (V5-TLR4) in the cellular environment.

### Codon optimization and *in silico* cloning

For the sake of maximal protein expression, the vaccine construct's codon was optimized by JCat (Java Codon Adaptation Tool) in *E. coli* (strain K12). The JCat resource provided the percentage of average GC content up to 70% and CAI value (0.95–1.0) of adapted sequences, which ensured a high expression rate of vaccine constructs in *E. coli*. The adapted codon sequences were introduced in the plasmid vector pET 28a (+) for the construction of recombinant plasmid sequences, which ensured expression and heterologous cloning in *E. coli*. Cloning of the vaccine DNA sequence provided a recombinant vector of 6,804 bp (Supplementary Figure S2).

### Immune simulation

**Figure 5:**
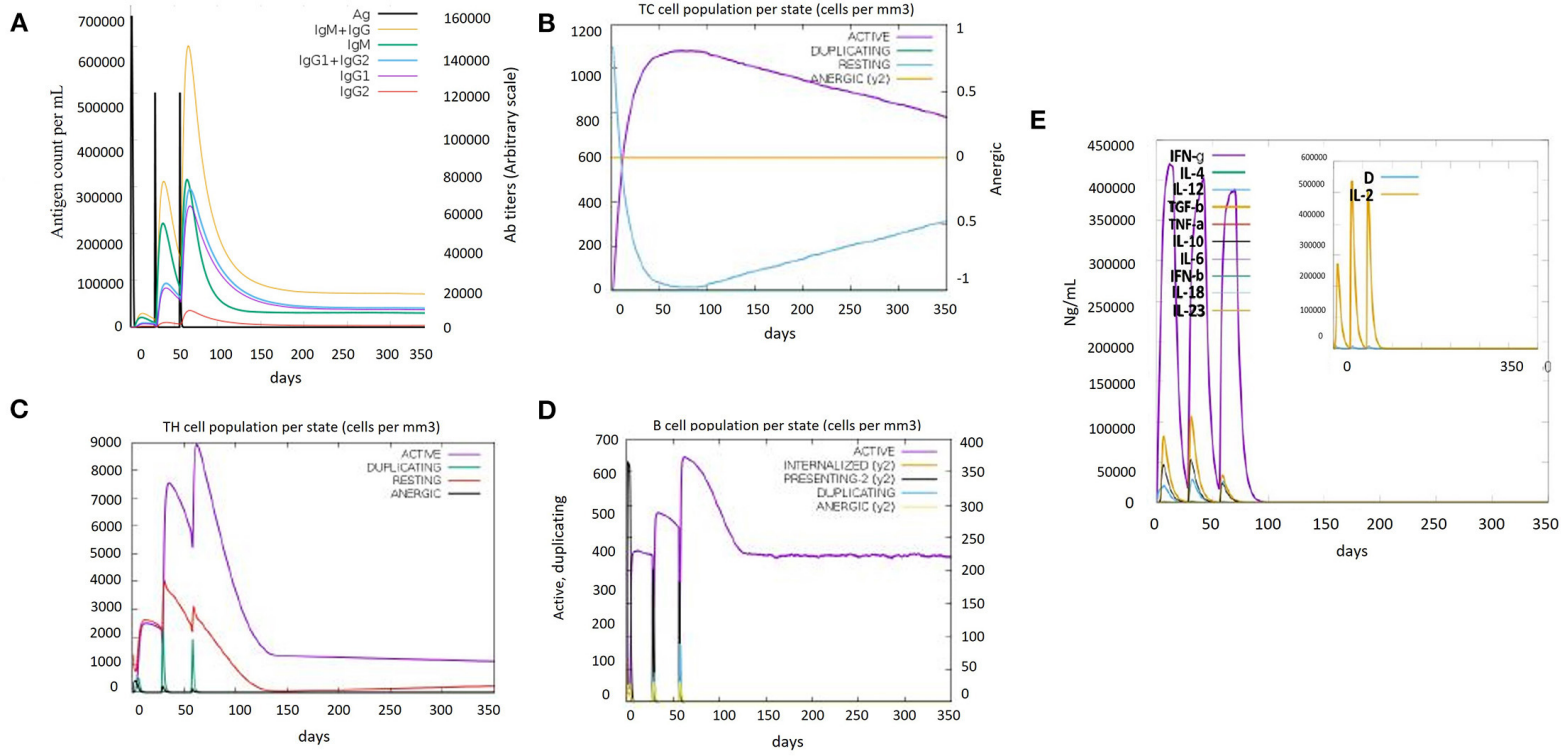
Immune simulation results of the designed vaccine construct (V5). **(A)** Immunoglobulin levels with respect to antigen concentration. **(B)** Cytotoxic T-cell population per state. **(C)** Helper T-cell population per state. **(D)** B-cell population per state. **(E)** Cytokine and interleukin levels after vaccine injection. The inset plot indicated high production of IL-2 along with a danger signal.

Immune simulations play a role in the development of an immune profile of the vaccine construct. The memory of immune cells was observed under C-ImmSim analysis to increase their half-life and successive immune responses by the cell (Bibi et al., 2021). Steadiness and real immune reactions were confirmed by the outcomes of this server. High IgM values indicated a primary immune response. Furthermore, the immunoglobulin expression level (IgG1+IgG2, IgM, and IgG+IgM) was increased, showing a decrease in antigen concentration (Figure 5A), which confirmed an increase in the active B-cell population (Figure 5D). Immunization led to an increase in the concentration of the active cytotoxic and helper T lymphocytes (Figures 5B, C). The cytokinin levels and IFN-gamma-inducing properties were also increased after every dose of the vaccine. Furthermore, the inset plot indicated that the chances of the danger caused by the vaccine are too low (Figure 5E). All the immune responses indicated that our vaccine is capable of causing a potent immune response.

## Discussion

*P. melaninogenica*, due to its irrefutable place within the CF respiratory microbiota in the pathophysiology of oral lichen planus (OLP) and periodontal diseases, has gained the attention of researchers to target this pathogen (Lamoureux et al., 2021). Although discrepancies in antimicrobial rates were observed, a constant increase in resistance is still trending, which provoked the researchers to look for an alternative to the current therapies. Identification of novel therapeutic drugs by targeting the core genes of bacterial species might be favorable. *In silico* approaches have become popular in designing novel drug and vaccine targets against pathogenic organisms with the help of bioinformatics.

While looking for an alternative drug target, subtractive proteomics or genomics were combined with reverse vaccinology to find a suitable vaccine target. In the present study, 14 complete genomes of *P. melaninogenica* were used to extract the core genes of the bacteria, which remain conserved throughout the species. The previous studies involved surveys and other experimental approaches to combat the infections of *P. melaninogenica*, but no *in silico* approaches were applied in the past. A total of 114 proteins were recognized as essential, virulent, antibiotic-resistant, and non-homologous, which could serve as novel drug or vaccine targets determined *by in silico* analysis.

PPI network analysis of these drug candidates filtered the hub proteins after predicting the subcellular location of proteins (Jalili et al., 2016). Druggable screening of the cytoplasmic proteins prioritized the non-homologs as novel discoveries in the identification of therapeutic drug targets against *P. melaninogenica*. A total of 18 proteins were prioritized as potential drug targets, of which 1 is KEGG-dependent and 17 are KEGG-independent (Table 2). Of these proteins, dihydroneopterin aldolase (ADK97401.1) is involved in the conversion of 7,8-dihydroneopterin to 6-hydroxymethyl-7,8-dihydropterin in the folate synthesis pathway of microorganisms. This pathway is very important for the growth and survival of many microorganisms. It was selected as a drug target because its inhibition can cause the death of microorganisms (Wang et al., 2006). Pyridoxal 5′-phosphate (PLP) (ADK95358.1) is an important coenzyme and is involved in a variety of reactions. The pyridoxal 5′-phosphate synthase is responsible for the synthesis of PLP. If PLP-synthase is inhibited in microorganisms, it leads to a decrease in their essential amino acids, leading to growth retardation or death of the microorganisms. Due to this reason, PLP-synthase was prioritized as a potential drug target (Strohmeier et al., 2006). Nicotinate phosphoribosyl transferase (NAPRT) (ADK95883.1) causes the conversion of nicotinic acid into nicotinamide adenine dinucleotide (NAD) (Audrito et al., 2020). NAD is involved in energy production and the repair of DNA in the event of any damage. If NAPRT is inhibited in bacteria, it can lead to many problems for them. It can also cause sensitivity to certain antibiotics (Ghanem et al., 2022). Glycosyltransferase (ADK95886.1), involved in the interconversion of sugars, was also prioritized as a drug target (Schmid et al., 2016). The putative phosphomethylpyrimidine kinase (ADK96110.1), the homolog of thiD, is involved in the initial stage of the biosynthesis of vitamin B1 (thiamin). Thiamin, in its activated form, thiamin diphosphate (ThDP), is very important for microorganisms. It is essential for many important metabolic processes, such as the breakdown of sugars and amino acids. Therefore, it was considered a drug target (Naz et al., 2019). Lipid A biosynthesis (KDO)2-(lauroyl)-lipid IVA acyltransferase (ADK96013.1) is responsible for the growth of bacterial cells at high temperatures. If this protein is deleted in a bacterium, it cannot survive at temperatures above 33°C (Zhou et al., 2021). Moreover, it is involved in the formation of Lipid A in bacteria, which is very important for the survival of the bacteria. If it is inhibited in bacteria, it can lead to the disruption of its outer membrane, which ultimately results in the death of the bacteria. Thus, it is a potential drug target against bacterial infections (Six et al., 2008). Another potential novel drug target is UvrD (ADK96570.1). It is a versatile protein found in microorganisms. Its role in DNA metabolism is crucial because it plays various functions such as repairing DNA mismatches during replication, participating in nucleotide excision repair and replication, promoting recombination by removing RecA filaments from DNA, and regulating transcription through interactions with RNA polymerase (Ordabayev et al., 2018). Overall, UvrD's involvement in maintaining DNA integrity and genomic stability is essential for the proper functioning of microorganisms (Ordabayev et al., 2018). Its inhibition can cause the death of microorganisms. Due to these reasons, it showed the potential to become a drug target. The gamma subunit of ATP synthase F1 (ADK96796.1) is essential for ATP production through oxidative phosphorylation in microorganisms (Xu et al., 2015). It plays a vital role in controlling ATP synthase activity and maintaining the proton gradient across the membrane. When the gamma subunit is inhibited, ATP synthesis decreases and the proton gradient is disrupted, which can impact various aspects of cellular metabolism, growth, and survival. Prolonged inhibition of ATP synthesis can ultimately result in cell death (Xu et al., 2015). Thus, targeting this important protein may be fatal for the pathogen and is an effective drug target. The novel drug targets, including aldolases, helicases, hydrolases, synthases, and transferases, investigated in this study have not been reported as drug targets in previous studies. According to the centrality–lethality rule, bacterial pathogens can be targeted by the inhibition or knockdown of these proteins.

To treat pathogenic infections, various antibiotics are discovered by using subtractive genomics or proteomics approaches, but there is still a demand to look for chimeric vaccine development due to increased antimicrobial resistance in clinical isolates of *P. melaninogenica*. Outer membrane proteins being involved in host–pathogen interaction were prioritized as potent vaccine targets in the reverse vaccinology approach (Lu et al., 2014). After analyzing different parameters, two vaccine candidates, i.e., reverse transcriptase (ADK95685.1) and hypothetical protein (ADK97014.1), were selected for the chimeric vaccine construction. Host cells are directly exposed to bacterial outer membrane proteins; hence, they are considered to be the most favorable candidate to be used in reverse vaccinology against invasive pathogens (Rizwan et al., 2017). Bacterial life and pathogenesis depend on these proteins as they play an important role in different functions such as nutrient acquisition, adhesion, and sustaining bacterial membrane integrity (Mishra et al., 2020).

B- and T-cell immunity is stimulated by antigenic regions of proteins in epitope mapping using the immunoinformatic approach, where the non-antigenic portion is excluded. Lead B- and T-cell overlapped epitopes were used to generate chimeric subunit constructs with different combinations of adjuvants and PADRE sequences for better immune responses and to overwhelm the HLA polymorphism throughout the population (Ghaffari-Nazari et al., 2015). Maximal expression of the prioritized vaccine construct (V5) was ensured by *in silico* cloning in pET 28a (+). A stable interaction between ligand and receptor was confirmed by molecular docking of the vaccine construct with human HLAs and TLR4, which showed the highest docking score. Response to antigens directly depends on the human body's defense system, which is fully equipped to respond. Cytokine and chemokine production to mediate cellular immune responses and the recognition of PAMPs (pathogen-associated molecular patterns) are controlled by TLRs, which are present on the surface of immune cells.

Innate and adaptive immunity responses might be possible with the designed vaccine, as inferred by the binding of V5 with TRL4. The stability and dynamic performance of docked complex V5-TLR4 were explored by molecular dynamic simulation, where steady binding of the complex was confirmed by an RMSD plot. Profound immune responses were expected, as predicted by immune simulation. Memory B and T cells were developed, and the production of helper T cells was evident from the results. Efficient Ig production was indicated by a high level of T cells, supporting a humoral response. Moreover, practical implementation of the designed lead vaccine might be advantageous to combat pathogenic diseases of *P. melaninogenica* in the near future, as inferred from *in silico* approaches.

## Conclusion

Before doing biological experiments, it is of utmost importance to depend on *in silico* approaches that can give a better idea about the probability and feasibility of discovering novel drug and vaccine targets. In the present study, *in silico* approaches such as subtractive proteomics and reverse vaccinology were applied to prospect proteins to serve them as drug or vaccine candidates from a conserved set of genes obtained from 14 complete genomes of *P. melaninogenica*. Several biological databases, comparative sequence analyses, and druggability analyses were performed to find the most potent drug candidate. Ultimately, 18 proteins were enlisted as prioritized druggable candidates to be addressed as novel targets against the bacterium that had not been reported in previous studies.

Two proteins were selected as vaccine candidates to develop a chimeric subunit vaccine. Maximal expression of the engineered vaccine construct (V5) was ensured by the *in silico* cloning of the chimeric construct in the bacterial system. Furthermore, the vaccine construct (V5) was docked with human immune cell receptors and TRL4 to confirm its stable binding and capability to stimulate cell-mediated immune responses. Steady binding of the docked complex was inferred by the molecular dynamic simulation analysis. Effective immunological memory to control *P. melaninogenica* infections was validated by immune simulation. All the proposed therapeutic targets further need validation in animal models. Various pathogenic strains of *P. melaninogenica* can be targeted by these therapeutic candidates as core genes were the basis of the study. The drug targets and the vaccine construct designed in this study deserve experimental validation to devise the proper treatment for the said pathogen.

## Data availability statement

The original contributions presented in the study are included in the article/Supplementary material, further inquiries can be directed to the corresponding authors.

## Author contributions

MSha: Conceptualization, Resources, Supervision, Writing—review and editing. AA: Formal analysis, Investigation, Writing—original draft. AQ: Formal analysis, Investigation, Writing—original draft. SJ: Formal analysis, Investigation, Methodology, Writing—original draft. AS: Formal analysis, Investigation, Writing—original draft. RU: Investigation, Methodology, Visualization, Writing—review and editing. EA: Funding acquisition, Visualization, Writing—review and editing. UN: Methodology, Visualization, Writing—review and editing. MShe: Formal analysis, Investigation, Writing—original draft. AZ: Formal analysis, Investigation, Methodology, Writing—original draft. SO: Funding acquisition, Resources, Writing—review and editing.

## Supplementary material

Supplementary Table S1 Analysis of MHC-II interacting epitopes (bold highlights the final epitopes).

Supplementary Table S2 Analysis of MHC-I interacting epitopes (bold highlights the final epitopes).

Supplementary Table S3 Selection of final vaccine constructs.

Supplementary Table S4 Physiochemical and general properties analysis of vaccine constructs.

Supplementary Figure S1 **(A)** Vaccine constructs V5 2D structure predicted by PRISIPRED server. **(B)** Vaccine constructs V5 2D structure predicted by PRISIPRED server.

Supplementary Figure S2 *In silico* restriction cloning of final vaccine construct (V5) into the *E. coli* pET28a (+) expression vector where red color shows the cloned vaccine construct.
