# Supplementary material for: Proteome level analysis of drug-resistant Prevotella melaninogenica for the identification of novel therapeutic candidates
Source: Front Microbiol. 2023 Sep 22;14:1271798. doi: 10.3389/fmicb.2023.1271798 (PMC10556700; doi:10.3389/fmicb.2023.1271798)
Supplement: Supplementary Table S1 — Analysis of MHC-II interacting epitopes (bold highlights the final epitopes). [file Table_1.DOCX]

**Table S1.** Analysis of MHC-II interacting epitopes (Bold highlights the final epitopes)

| **Protein IDs** | **Allele** | **Start** | **End** | **Predicted Epitopes** | **Rank** | **Antigenicity** | **Conservancy** | **IFN Inducers** | **IL4 inducer** | **IL10 inducer** |
| --- | --- | --- | --- | --- | --- | --- | --- | --- | --- | --- |
| ADK95685.1 | **HLA-DRB1*04:01** | **46** | **60** | **RGKQLSFLADTNNVQ** | **0.37** | **Antigen** | **80.00%** | **No data** | **Non- inducer** | **Non-inducer** |
|  | **HLA-DRB1*04:04** | **82** | **96** | **KGSLRGILNALNILL** | **0.27** | **Antigen** | **60.00%** | **Positive** | **Non- inducer** | **Non-inducer** |
|  | **HLA-DQA1*01:02/DQB1*06:02** | **328** | **342** | **RGKLNYMKMVRGADD** | **0.42** | **Antigen** | **86.67%** | **Positive** | **Non- inducer** | **Non-inducer** |
|  | HLA-DRB4*01:01 | 423 | 437 | MVIILVAIILIKLFL | 0.02 | Antigen | 46.67% | Positive | Non- inducer | Non-inducer |
| ADK97014.1 | **HLA-DRB5*01:01** | **354** | **368** | **YRAGFSYASPYLKIN** | **0.02** | **Antigen** | **86.67%** | **No data** | **Inducer** | **Inducer** |
|  | HLA-DRB1*01:01 | 6 | 20 | AAAFLAGLAVQVSAQ | 0.14 | Antigen | 93.33% | Negative | Non-inducer | Non-inducer |
|  | **HLA-DRB3*02:02** | **413** | **427** | **ENMFRINVGFTFNER** | **0.17** | **Antigen** | **93.33%** | **Positive** | **Inducer** | **Non-inducer** |
|  | **HLA-DRB3*01:01** | **210** | **224** | **AQVKSYKVDFGAQYT** | **0.18** | **Antigen** | **86.67%** | **Positive** | **Inducer** | **Non-inducer** |
|  | **HLA-DRB3*01:01** | **71** | **85** | **DSLSFIFDAGVSLQL** | **0.23** | **Antigen** | **100.00%** | **Positive** | **Non-inducer** | **Non-inducer** |
|  | **HLA-DRB3*02:02** | **280** | **294** | **FGVGLMWNHNNRLKF** | **0.33** | **Antigen** | **86.67%** | **Positive** | **Inducer** | **Inducer** |
|  | **HLA-DRB1*13:02** | **199** | **213** | **SYVNTLSKNYSAQVK** | **0.42** | **Antigen** | **93.33%** | **Positive** | **Inducer** | **Inducer** |
|  | **HLA-DRB3*02:02** | **265** | **279** | **TTRYVVSNSLELPHT** | **0.87** | **Antigen** | **86.67%** | **Positive** | **Inducer** | **Inducer** |
|  | **HLA-DRB1*09:01** | **373** | **387** | **PRELSASLGVGIPIL** | **1.1** | **Antigen** | **93.33%** | **Positive** | **Non- inducer** | **Inducer** |
|  | HLA-DRB1*09:01 | 105 | 119 | VVASFRAFKHVGVSF | 0.24 | Non-antigen | 100.00% | No data | Non- inducer | Non-inducer |
