# Supplementary material for: Proteome level analysis of drug-resistant Prevotella melaninogenica for the identification of novel therapeutic candidates
Source: Front Microbiol. 2023 Sep 22;14:1271798. doi: 10.3389/fmicb.2023.1271798 (PMC10556700; doi:10.3389/fmicb.2023.1271798)
Supplement: Supplementary Table S2 — Analysis of MHC-I interacting epitopes (bold highlights the final epitopes). [file Table_2.DOCX]

**Table S2.** Analysis of MHC-I interacting epitopes (Bold highlights the final epitopes)

| **Protein IDs** | **Start** | **End** | **Predicted Epitopes** | **Alleles** | **Predicted score** | **Rank** | **Antigenicity** | **Toxicity** | **Immunogenicity** | **Conservancy** |
| --- | --- | --- | --- | --- | --- | --- | --- | --- | --- | --- |
| ADK95685.1 | 21 | 29 | RHLSDAQEL | HLA-B*39:01 | 0.821535 | 0.056 | Non-Antigen | Non-Toxin | -0.1509 | 55.56% |
|  | 44 | 52 | DFRGKQLSF | HLA-B*08:01 | 0.57656 | 0.157 | Antigen | Non-Toxin | -0.37416 | 77.78% |
|  | 97 | 105 | QTYDEPTPW | HLA-B*58:01 | 0.969825 | 0.031 | Non-Antigen | Non-Toxin | 0.13446 | 88.89% |
|  | **125** | **133** | **KRYILNLDL** | **HLA-B*27:05** | **0.912516** | **0.06** | **Antigen** | **Non-Toxin** | **0.11943** | **100.00%** |
|  | 141 | 149 | TRQQVADCL | HLA-B*39:01 | 0.631382 | 0.137 | Antigen | Non-Toxin | -0.08991 | 88.89% |
|  | 151 | 159 | AEPFGFSSL | HLA-B*40:01 | 0.860905 | 0.122 | Non-Antigen | Non-Toxin | 0.02112 | 88.89% |
|  | 162 | 170 | KLISGLATV | HLA-A*02:01 | 0.983689 | 0.007 | Non-Antigen | Non-Toxin | -0.04501 | 88.89% |
|  | 184 | 192 | ATSPTLSNF | HLA-B*58:01 | 0.855327 | 0.129 | Antigen | Non-Toxin | -0.1809 | 88.89% |
|  | **207** | **215** | **AQGITFTRY** | **HLA-B*15:01** | **0.894992** | **0.049** | **Antigen** | **Non-Toxin** | **0.35592** | **88.89%** |
|  | **237** | **245** | **QVKAIVERY** | **HLA-A*26:01** | **0.840721** | **0.025** | **Antigen** | **Non-Toxin** | **0.25257** | **88.89%** |
|  | **248** | **256** | **RLNEEKTHL** | **HLA-A*02:01** | **0.771464** | **0.126** | **Antigen** | **Non-Toxin** | **0.04481** | **88.89%** |
|  | **261** | **269** | **RRQEVTGLM** | **HLA-B*27:05** | **0.808352** | **0.142** | **Antigen** | **Non-Toxin** | **0.16201** | **100.00%** |
|  | 279 | 287 | RYVREIRSL | HLA-A*24:02 | 0.828191 | 0.089 | Non-Antigen | Non-Toxin | 0.23528 | 88.89% |
|  | 305 | 313 | KSYRQQHGK | HLA-A*03:01 | 0.915162 | 0.035 | Antigen | Non-Toxin | -0.12386 | 66.67% |
|  | 350 | 358 | SRYTSLQQR | HLA-B*27:05 | 0.924111 | 0.052 | Antigen | Non-Toxin | -0.29912 | 77.78% |
|  | 366 | 374 | VAYKAYMGK | HLA-A*03:01 | 0.763618 | 0.152 | Antigen | Non-Toxin | -0.31198 | 77.78% |
|  | 382 | 390 | DRMTSANVL | HLA-B*39:01 | 0.858694 | 0.036 | Antigen | Non-Toxin | -0.12355 | 33.33% |
|  | 415 | 423 | KRILNFIVM | HLA-B*27:05 | 0.857211 | 0.104 | Antigen | Non-Toxin | 0.27238 | 44.44% |
|  | 432 | 440 | LIKLFLKSL | HLA-B*08:01 | 0.697445 | 0.096 | Antigen | Non-Toxin | -0.25626 | 33.33% |
| ADK97014.1 | 34 | 42 | ALASQATSF | HLA-B*15:01 | 0.928251 | 0.023 | Antigen | Non-Toxin | -0.29364 | 88.89% |
|  | 19 | 27 | AQSGTNSPY | HLA-B*15:01 | 0.938479 | 0.02 | Antigen | Non-Toxin | -0.13399 | 100.00% |
|  | 75 | 83 | FIFDAGVSL | HLA-A*02:01 | 0.89711 | 0.051 | Non-Antigen | Non-Toxin | 0.0685 | 100.00% |
|  | **9** | **17** | **FLAGLAVQV** | **HLA-A*02:01** | **0.972513** | **0.012** | **Antigen** | **Non-Toxin** | **0.03999** | **88.89%** |
|  | **416** | **424** | **FRINVGFTF** | **HLA-B*27:05** | **0.956101** | **0.024** | **Antigen** | **Non-Toxin** | **0.23027** | **100.00%** |
|  | **175** | **183** | **FSFGANIGY** | **HLA-A*26:01** | **0.677038** | **0.083** | **Antigen** | **Non-Toxin** | **0.23623** | **88.89%** |
|  | 358 | 366 | FSYASPYLK | HLA-A*03:01 | 0.830731 | 0.093 | Antigen | Non-Toxin | -0.14297 | 77.78% |
|  | 372 | 380 | GPRELSASL | HLA-B*07:02 | 0.995498 | 0.004 | Antigen | Non-Toxin | -0.11262 | 100.00% |
|  | **325** | **333** | **GQFNDRHKF** | **HLA-B*15:01** | **0.916664** | **0.03** | **Antigen** | **Non-Toxin** | **0.00311** | **88.89%** |
|  | 278 | 286 | HTFGVGLMW | HLA-B*58:01 | 0.992681 | 0.005 | Non-Antigen | Non-Toxin | 0.03224 | 88.89% |
|  | **123** | **131** | **PYTNVGYNF** | **HLA-A*24:02** | **0.950718** | **0.019** | **Antigen** | **Non-Toxin** | **0.07129** | **100.00%** |
|  | 394 | 402 | SMLNISAEW | HLA-B*58:01 | 0.799705 | 0.174 | Non-Antigen | Non-Toxin | 0.05528 | 88.89% |
|  | 192 | 200 | STNTYSDSY | HLA-A*01:01 | 0.929203 | 0.038 | Antigen | Non-Toxin | -0.20031 | 88.89% |
|  | 142 | 150 | STSSVNATY | HLA-A*26:01 | 0.848784 | 0.023 | Antigen | Non-Toxin | -0.13036 | 88.89% |
|  | 225 | 233 | YAVDKKNEL | HLA-B*08:01 | 0.741637 | 0.075 | Antigen | Non-Toxin | -0.32424 | 77.78% |
|  | **104** | **112** | **YVVASFRAF** | **HLA-A*26:01** | **0.684668** | **0.08** | **Antigen** | **Non-Toxin** | **0.06841** | **100.00%** |
