# Supplementary material for: Proteome level analysis of drug-resistant Prevotella melaninogenica for the identification of novel therapeutic candidates
Source: Front Microbiol. 2023 Sep 22;14:1271798. doi: 10.3389/fmicb.2023.1271798 (PMC10556700; doi:10.3389/fmicb.2023.1271798)
Supplement: Supplementary Table S3 — Selection of final vaccine constructs. [file Table_3.DOCX]

**Table S3.** Selection of final vaccine constructs.

| **Vaccine constructs** | **Nucleotides** | **CAI values** | **GC content (%)** | **SOLpro** | **AntigenPro** | **VaxiJen 2.0** | **Allergenicity** |
| --- | --- | --- | --- | --- | --- | --- | --- |
| V1 | 1494 | 0.97 | 50.13 | 0.702572 | 0.86988 | 0.668 | Non-allergen |
| V2 | 1608 | 0.96 | 52.3 | 0.835978 | 0.894787 | 0.66 | Non-allergen |
| V3 | 1581 | 0.95 | 51.8 | 0.900272 | 0.844358 | 0.663 | Non-allergen |
| V4 | 1266 | 0.96 | 50.95 | 0.564514 | 0.894169 | 0.695 | Non-allergen |
| **V5** | **1431** | **0.98** | **49.41** | **0.923653** | **0.856118** | **0.764** | **Non-allergen** |
| V6 | 1545 | 0.97 | 51.72 | 0.957372 | 0.813796 | 0.749 | Non-allergen |
| V7 | 1518 | 0.96 | 51.19 | 0.971729 | 0.802764 | 0.753 | Non-allergen |
| **V8** | **1203** | **0.97** | **50.12** | **0.920178** | **0.854637** | **0.81** | **Non-allergen** |
