# Supplementary material for: Proteome level analysis of drug-resistant Prevotella melaninogenica for the identification of novel therapeutic candidates
Source: Front Microbiol. 2023 Sep 22;14:1271798. doi: 10.3389/fmicb.2023.1271798 (PMC10556700; doi:10.3389/fmicb.2023.1271798)
Supplement: Supplementary Table S4 — Physiochemical and general properties analysis of vaccine constructs. [file Table_4.DOCX]

**Table S4.** Physiochemical and General properties analysis of vaccine constructs.

| **Vaccine Constructs** | **No. of amino acids** | **Molecular weight (KDa)** | **PI** | **Aliphatic index** | **Hydropathicity (GRAVY)** | **Instability index** |
| --- | --- | --- | --- | --- | --- | --- |
| V1 | 498 | 52 | 5.66 | 73.05 | -0.313 | 20.77 |
| V2 | 536 | 58 | 5.54 | 70.82 | -0.442 | 27.05 |
| V3 | 527 | 57 | 5.41 | 72.56 | -0.422 | 29.86 |
| V4 | 422 | 45 | 9.07 | 62.8 | -0.449 | 22.57 |
| **V5** | **477** | **50** | **8.5** | **70.46** | **-0.352** | **19.81** |
| V6 | 515 | 56 | 7.9 | 73.88 | -0.484 | 26.42 |
| V7 | 506 | 55 | 6.8 | 75.75 | -0.464 | 29.34 |
| **V8** | **401** | **43** | **9.6** | **66.31** | **-0.503** | **21.52** |
