# Supplementary figures and images for: Proteome level analysis of drug-resistant Prevotella melaninogenica for the identification of novel therapeutic candidates
Source: Front Microbiol. 2023 Sep 22;14:1271798. doi: 10.3389/fmicb.2023.1271798 (PMC10556700; doi:10.3389/fmicb.2023.1271798)

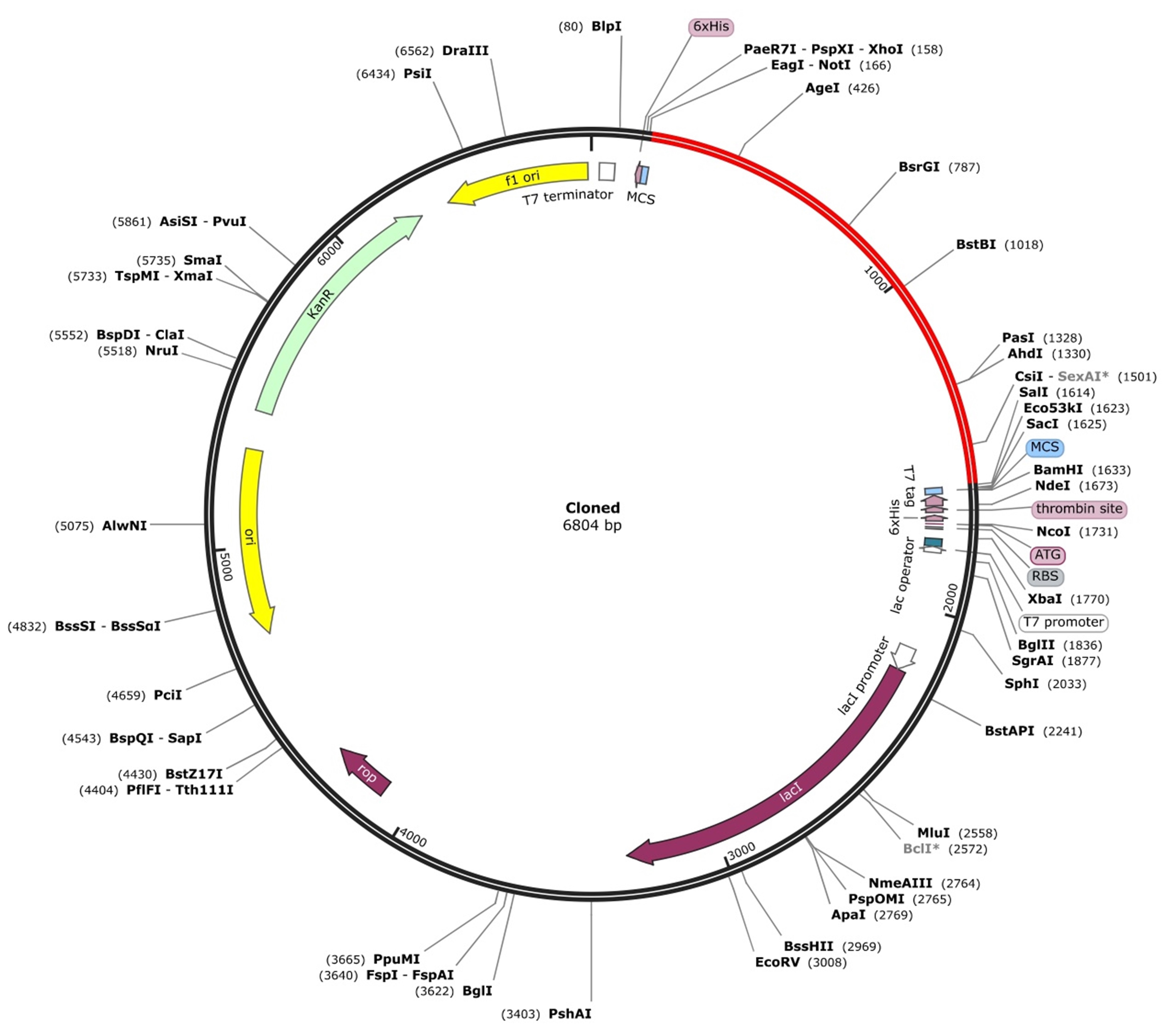

Supplement: Supplementary Figure S2 — In silico restriction cloning of final vaccine construct (V5) into the E. coli pET28a (+) expression vector where red color shows the cloned vaccine construct. [file Image_2.JPEG]
